# Long-term sensorimotor changes after a sciatic nerve block with bupivacaine and liposomal bupivacaine in a high-fat diet/low-dose streptozotocin rodent model of diabetes

**DOI:** 10.3389/fanes.2024.1422353

**Published:** 2024-06-05

**Authors:** Susanna C. Byram, Krista M. Lotesto, Michael Volyanyuk, Jacob E. Exline, Elizabeth A. Sager, Eileen M. Foecking

**Affiliations:** 1 Department of Anesthesiology and Perioperative Medicine, Loyola University Chicago Medical Center, Maywood, IL, United States; 2 Stritch School of Medicine, Loyola University Chicago Medical Center, Maywood, IL, United States; 3 Department of Research and Development Services, Edward Hines Jr. VA Hospital, Hines, IL, United States,; 4 Surgical Service, Edward Hines Jr. VA Hospital, Hines, IL, United States,; 5 The Burn and Shock Trauma Research Institute, Loyola University Chicago Medical Center, Maywood, IL, United States; 6 Neuroscience Graduate Program, Loyola University Chicago Stritch School of Medicine, Maywood, IL, United States; 7 Department of Otolaryngology, Head and Neck Surgery, Loyola University Chicago Medical Center, Maywood, IL, United States; 8 Department of Molecular Pharmacology and Neuroscience, Loyola University Chicago Stritch School of Medicine, Maywood, IL, United States

**Keywords:** regional anesthesia, local anesthetic toxicity, diabetic peripheral neuropathy, sciatic nerve block, peripheral nerve injury, bupivacaine, liposomal bupivacaine, neuropathic pain

## Abstract

**Introduction:**

It is unclear whether patients with diabetes are more susceptible to nerve toxicity of local anesthetics or whether nerve blocks can accelerate the progression of diabetic peripheral neuropathy. Bupivacaine is one of the most widely used local anesthetics for regional anesthesia despite many pre-clinical studies demonstrating neurotoxicity. Herein, we report the long-term functional consequences of sciatic nerve block with bupivacaine and liposomal bupivacaine (Exparel^®^) in an animal model of diabetes.

**Methods:**

Male Sprague Dawley rats were subject to standard chow/vehicle or high-fat diet/low-dose streptozotocin to induce a diabetic phenotype. Animals were then subdivided into groups that received repeated sciatic nerve blocks of saline, bupivacaine, or liposomal bupivacaine. Mechanical allodynia and thermal hyperalgesia were assessed prior to and 12 weeks following nerve blocks utilizing the von Frey and Hargreaves tests, respectively. Exploratory and locomotor activity were assessed with open field testing, and nerve conduction velocity testing was conducted prior to the termination of the study at 28 weeks.

**Results:**

Animals in the diabetic group developed sustained hyperglycemia >200 mg/dl and signs of peripheral neuropathy six weeks after treatment with streptozotocin, which persisted until the end of the study. Twelve weeks after a repeated sciatic nerve block with saline, bupivacaine, or liposomal bupivacaine, results indicate significant interaction effects of the disease group (control vs. diabetic) and local anesthetic treatment. Overall, diabetic status resulted in worse sensorimotor function compared to control animals. Treatment with perineural bupivacaine resulted in worse sensorimotor functions in both control and diabetic animals. Furthermore, bupivacaine treatment in diabetic animals with pre-existing neuropathy exacerbated sensorimotor function in some measures. In contrast, liposomal bupivacaine did not appear to cause any negative effects on functional outcomes for control or diabetic animals.

**Conclusion:**

Our data indicate that bupivacaine, and not liposomal bupivacaine, causes long-term changes in tactile allodynia, thermal hyperalgesia, locomotor behaviors, and nerve conduction velocity in control as well as a high-fat diet/low-dose streptozotocin rodent model of diabetes. These results highlight the necessity to investigate safe peripheral nerve block strategies to preserve long-term functional independence in patients with or at risk for diabetic peripheral neuropathy.

## 1 Introduction

The escalating burden of type 2 diabetes mellitus and its accompanying morbidities is a significant concern in healthcare. According to the International Diabetes Federation, over 530 million adults live with diabetes worldwide. The incidence is expected to rise above 750 million adults by 2,045, accounting for an estimated annual expenditure of almost 1 trillion US dollars (1). Accordingly, diabetes is present in an escalating number of patients presenting for surgical procedures, prompting a critical need to optimize perioperative care for this growing population (2–4). While patients with diabetes are known to have increased complications in the immediate post-operative period compared to non-diabetic patients, long-term complications, such as worsening of peripheral neuropathy, have not been studied (5). Approximately 50% of patients with diabetes will be affected by peripheral neuropathy in their lifetime (6). The sensorimotor impairments of diabetic peripheral neuropathy are progressive and can lead to debilitating chronic pain, poor functional status, and impaired quality of life (6). Diabetes-related peripheral neuropathy places patients at increased risk of developing foot injuries and infections, which can ultimately result in lower extremity amputation (6, 7). Due to the gravity of such debilitating outcomes, measures to safeguard against the worsening of peripheral neuropathy should be taken in the perioperative period.

Perioperative pain control is a critical component of patient care. Adequate pain control after surgery contributes to decreased morbidity, increased quality of life, and patient satisfaction (8, 9). By using local anesthetics for nerve block procedures, regional anesthesia improves perioperative pain management and has become an essential component for enhanced recovery after surgery (10). In addition, the use of perioperative nerve blocks can reduce the need for general anesthesia and decrease the use of systemic opioids (8, 9). While infrequent, adverse neurologic outcomes have been linked to local anesthetic use (8, 11, 12). Clinical dogma has purported that patients with pre-existing nerve dysfunction, including diabetic peripheral neuropathy, are more susceptible to toxicity from local anesthetics and subsequent risk for worsening neuropathy after a peripheral nerve block (13–17). Bupivacaine is one of the most used local anesthetics for peripheral nerve blocks. However, studies using bupivacaine have reported cellular toxicity (18–22). Additionally, we have previously reported that bupivacaine, but not liposomal bupivacaine, exacerbates motoneuron death and delays functional recovery in a peripheral nerve injury model, suggesting that pre-existing nerve injury increases the risk of bupivacaine toxicity (23, 24). Others have described delayed motor and sensory recovery and increased nerve damage after a sciatic nerve block using bupivacaine in diabetic patients and/or animals (16, 25–29). Recently, novel local anesthetic formulations, such as liposomal bupivacaine, are being developed and may also be less toxic to nerves. Several studies report that liposomal bupivacaine causes less cellular toxicity than standard bupivacaine and postulate the slow release and longer time to peak concentration may account for this difference (30, 31). The long-term consequences of perineural local anesthetics in patients with diabetic peripheral neuropathy are unknown (32), and there is no expert consensus on the safest practice regarding peripheral nerve blocks in patients with diabetes. Therefore, a better understanding of the effects of commonly used nerve block anesthetics should be prioritized to inform the development of safe peripheral nerve block strategies and preserve long-term functional independence in patients with or at risk for diabetic peripheral neuropathy.

We employed the high-fat diet/low-dose streptozotocin rodent model of diabetes to study the functional consequences of repeated sciatic nerve block with bupivacaine and liposomal bupivacaine. Outcome measures included mechanical allodynia, thermal hyperalgesia, exploratory and locomotor behaviors, and nerve conduction velocity. We hypothesized that perineural bupivacaine, but not liposomal bupivacaine, would exacerbate the severity of sensorimotor dysfunction in an animal model of diabetic peripheral neuropathy.

## 2 Materials and methods

### 2.1 Animals

**FIGURE 1:**
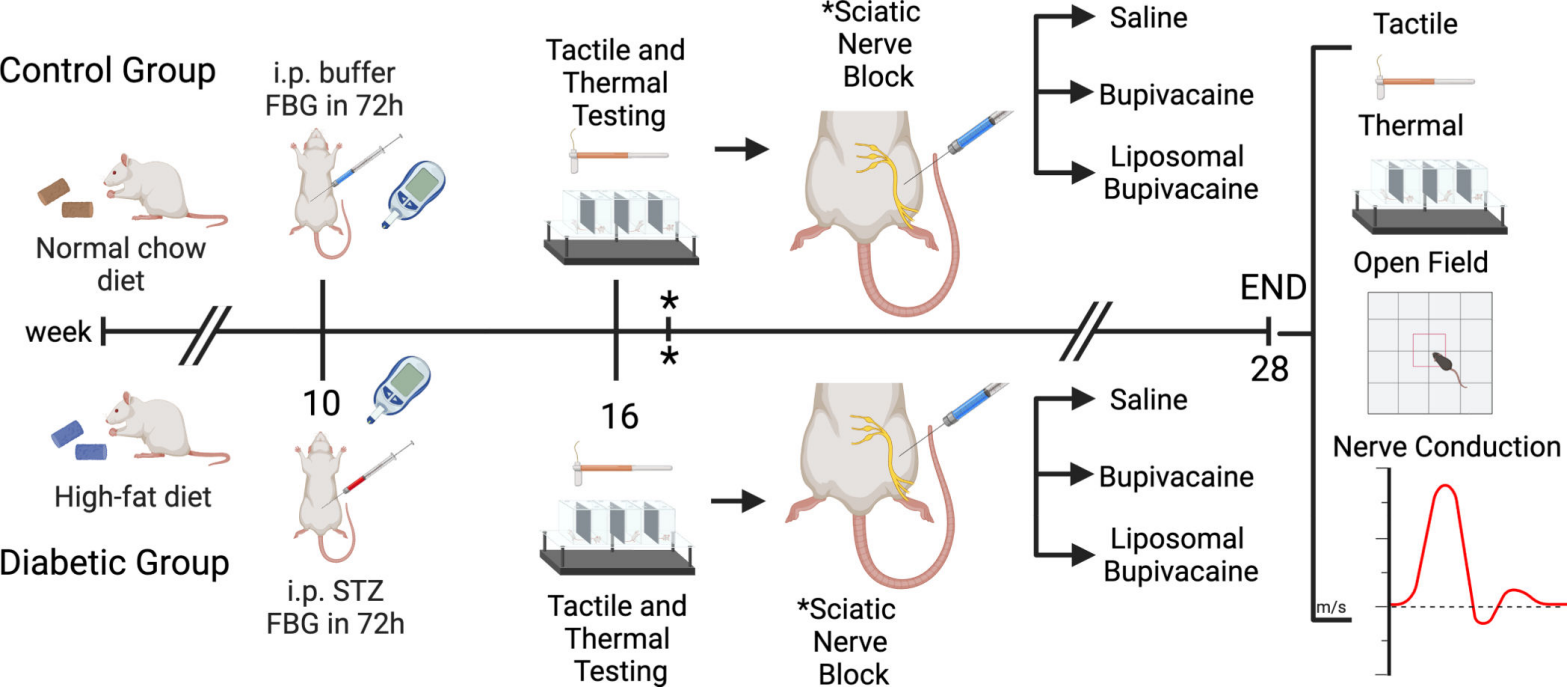
Experimental design. Male Sprague-Dawley rats were divided into two groups (*n* = 36 per group). The control group was fed a standard rat chow, the diabetic group was fed a high-fat diet, and the animals remained on their assigned diet for the 28-week study period. After 10 weeks of diet exposure, animals in the diabetic group were intraperitoneally (i.p) injected with a low dose of streptozotocin. Animals in the control group were given a citrate buffer vehicle. Diabetes (hyperglycemia) was confirmed by measuring fasting blood glucose (FBG) levels 1 week after injection. Only animals with a fasting blood glucose greater than 200 mg/dl were considered diabetic and included in the study. At week 16 (six weeks after i.p. treatments), tactile allodynia and thermal hyperalgesia were tested to confirm the development of peripheral neuropathy, followed by a percutaneous sciatic nerve block using saline, bupivacaine, or liposomal bupivacaine. * Sciatic nerve block was repeated 1 week later. At week 28 (12 weeks after sciatic nerve blocks), tactile and thermal sensitivity tests, exploratory and locomotor behaviors (via open-field analysis), and nerve conduction velocity were performed. Figure created with BioRender.com.

All animals were housed and manipulated according to institutional and National Institutes of Health guidelines, and the experimental procedures described were approved by the Animal Care and Use Committee of Edward Hines Jr., VA Hospital (Hines, IL). A graphical representation of the experimental design is represented in Figure 1. A total of 72 male Sprague-Dawley outbred rats (200–220 g) were obtained from Harlan Teklad (Madison, WI), kept under a 12-h light/dark cycle, and provided food and water *ad libitum* unless otherwise stated. Animals were permitted one week to acclimate to their environment, followed by one week of handling and behavioral equipment acclimation. Upon arrival, all animals were provided standard rat chow during the acclimation and baseline testing. After the acclimation periods, animals were divided into two groups (*N* = 36 per group). The control group was fed a standard rat chow (6.2% kcal fat, 44% kcal carbohydrate, 18% protein, Envigo Teklad Rodent Diet 2018SCDiet 2018SC, Madison, WI), and the diabetic group was fed a high-fat diet (60% kcal fat, 21% kcal carbohydrate, 18% protein, Envigo Teklad TD.06414, Madison, WI). Animals remained on their assigned diet for the remainder of the 28-week study period. After 10 weeks of diet exposure, animals in the diabetic group were given a low dose of streptozotocin (30 mg/kg in 0.1M citrate buffer, pH 4.4; Millipore Sigma S0130, St. Louis, MO) via intraperitoneal injection to induce hyperglycemia. The control group was given a citrate buffer vehicle only. Diabetes (hyperglycemia) was confirmed by measuring fasting blood glucose levels using an AlphaTrak glucometer (Zoetis US, Parsipanny, NJ) 1 week after injecting streptozotocin. Only animals with a fasting blood glucose greater than 200 mg/dl were considered diabetic and included in the study. Animals were then observed for 6 weeks on their respective diets to allow the development of diabetic peripheral neuropathy. Tactile allodynia and thermal hyperalgesia were tested, as described below, to confirm the development of peripheral neuropathy.

### 2.2 Repeated percutaneous sciatic nerve block

At week 16 (6 weeks after streptozotocin/vehicle injection), the control and diabetic groups were further subdivided into three sciatic nerve block treatment sub-groups as follows: saline (*N* = 12), bupivacaine (*N* = 12), and liposomal bupivacaine (*N* = 12). We performed a percutaneous sciatic nerve block with the assigned treatment, using methods described by Thalhammer et al. (33) and Kroin et al. (25, 33). Animals were briefly anesthetized with 3% isoflurane and placed in a left lateral recumbent position. A 25-gauge Stimuplex^®^ D Insulated Needle (B-Braun Medical Inc., Bethlehem, PA), with a pre-filled syringe attached, was inserted percutaneously into the sciatic notch between the greater trochanter and the ischial tuberosity pointing toward the ischium. Stimulating pulses (0.2 mA, 2 ms, 1 Hz) were delivered using a nerve stimulator (Stimuplex^®^ HNS 12; B-Braun, Bethlehem, PA), and the needle advanced until a vigorous ipsilateral hind-leg kick was observed, indicating proximity to the sciatic nerve. Then, 0.6 ml of 0.9% normal saline, 0.5% bupivacaine HCl, or 13.3% liposomal bupivacaine (Exparel^®^, Pacira Pharmaceuticals, Parsippany, NJ) was slowly injected over 5 s (s). A successful nerve block was confirmed 30 and 60 min after injection by observing the presence or absence of toe-spreading when the animal was gently lifted. Behavior was recorded as present or absent. One week later, the percutaneous sciatic nerve block was repeated in each animal. The animal was removed from the study if a nerve block was unsuccessful.

### 2.3 Outcome measures

Tactile and thermal sensitivity tests were assessed utilizing the von Frey and Hargreaves methods just before the first sciatic nerve block and again 12 weeks after the block. In addition, exploratory and locomotor behaviors were assessed with open-field analysis and nerve conduction velocity was performed 12 weeks after the block. All tests were performed by an investigator blinded to the treatment group.

*Tactile allodynia* was evaluated by calculating the 50% force paw withdrawal threshold using manual Von Frey filaments via the classic up-down method (34). Briefly, animals were placed on a mesh testing platform with separated enclosures and allowed to acclimate for 15 min. A logarithmic series of Von Frey filaments with target forces of 1.4 g, 2 g, 4 g, 6 g, 8 g, 10 g, 15 g, 26 g, and 60 g were individually applied perpendicularly to the hind paw plantar surface for 5 s in an ascending or descending manner (Aesthesio #37450–275, Ugo Basile, Varese, Italy). If the animal displayed a positive response, defined as a quick withdrawal or lifting of the hind paw, the next descending force filament would follow. If the animal displayed a negative response, defined as the absence of hind paw withdrawal or lifting, the next ascending force filament would follow. A minimum of 4 fiber presentations were completed after the first change in direction, up to 9 total filament presentations. The 50% force paw withdrawal threshold was calculated using an online algorithm created by Christensen and colleagues (35, 36).

*Thermal sensitivity* was determined using the Hargreaves method and apparatus (Ugo Basile Thermal Plantar Analgesia Instrument-37370, Varese, Italy (37);. Animals were placed onto the plexiglass testing platform with separated enclosures and allowed to acclimate for 15 min. The thermal threshold was assessed by measuring the hind paw withdrawal latency to an infrared heat stimulus. The hind paw withdrawal latency is automatically detected and recorded in seconds (s) from the onset of the heat stimulus (intensity 70%, 30-s cutoff time) until paw withdrawal. Five recordings were taken for each hind paw, with the fastest and slowest withdrawal times discarded for each hind paw. A mean latency was calculated per animal.

*Open field test (OFT)* was used to evaluate locomotor activity. Animals were acclimated to the testing room for 15 min before testing. Animals were then placed into the center of an opaque, gray-colored, 60 cm × 60 cm arena with 40.5 cm walls. Each subject was initially placed into the center of the arena, facing the wall designated as North. Activity was recorded for 10 min and analyzed to assess motor activity and exploration behavior. Noldus EthoVision XT 15 software was used to process the recorded tracks using LOWESS smoothing (*h* = 10) and generate locomotor activity data. Time spent moving, time spent highly mobile, total distance traveled, mean velocity, mean acceleration, and number of rears were analyzed. Movement was defined as when the center point of the animal was moving continuously with a start velocity of ≥2.00 cm/s and stop velocity < 1.75 cm/s. Mobility measures the percent change of the animal’s body area. “Highly mobile” was defined as a greater than 5% change in body contour. The distance traveled, velocity, and acceleration were filtered to segments when the animal progressively moved, as defined above. The open-source Behavioral Observation Research Interactive Software (BORIS) was used to manually count the number of rears (38). A rear was defined as the animal’s forepaws leaving the floor. Finally, heatmaps and tracings of the nose and center points were used to qualitatively describe the animal’s movement and location preferences.

*Motor nerve conduction velocity (NCV)* was evaluated at the end of the study. At the conclusion of the study, sciatic nerve conduction velocity was determined under isoflurane anesthesia using an electromyogram system (TECA Synergy, Viasys Healthcare, Conshohocken, Pennsylvania). Animals were anesthetized with 3% isoflurane, and normothermia was maintained with a heating pad. A stimulating electrode was inserted at the sciatic notch and at the Achilles tendon. The sciatic nerve was stimulated (0.2 ms, 1.0 Hz, supramaximal intensity), and the evoked potential was recorded by a pin electrode placed in the intraosseous muscle in the plantar pad of the hind limb. The M-wave was measured for each animal as an average of 10 individual evoked responses, repeated in triplicate, and then averaged per animal. Nerve conduction velocity was then calculated as the distance from the notch to the ankle divided by the difference in onset latency to the evoked responses.

### 2.4 Sample size, randomization, and blinding

The target sample size for evoked responses was 8 per group based on a power analysis and expected attrition to 6 per group. A power analysis shows that the sample size of 6 has a 80% power to detect an effect size of 2.0 units, assuming a 5% significance level and a two-sided test. For open field tests, a power analysis shows that the sample size of 4 has 80% power to detect an effect size of 2.4, assuming a 5% significance level and a two-sided test. Data are combined from experiments with two cohorts of animals for all experimental data, except for locomotor behavior, which was only measured in one cohort. Rats were randomly assigned to diet and treatment groups at the beginning of the study. Efforts to blind the study included medications prepared and coded by a separate investigator, behavioral analysis conducted by a blinded investigator, and keeping data blinded until analysis.

### 2.5 Statistical analysis

Exclusion criteria were established *a priori*. Animals were removed from the study if (1) signs of illness or injury could affect behavioral testing, (2) fasting blood glucose was <200 mg/dl in the diabetic group, (3) a sciatic nerve block was unsuccessful as determined by the presence of the toe-spreading reflex after treatment, or (4) premature death. Data were analyzed using IBM SPSS 29, and graphically represented using GraphPad Prism 10 (San Diego, CA, USA). Data analysis included repeated-measures 2-way analysis of variance (ANOVA) followed by Tukey’s HSD multiple comparisons test to compare weight and fasting blood glucose over time. Unpaired *t*-tests compared the mean evoked response measures (tactile and thermal) between control and diabetic animals before sciatic nerve blocks. After nerve blocks were performed, two-way ANOVAs followed by Tukey’s HSD multiple comparisons tests were conducted to examine the effects and/or interaction of diabetic status and local anesthetic treatment. Probability values less than 0.05 ( *p* < 0.05) were considered statistically significant, and data are expressed as mean ± SEM unless otherwise stated. If an interaction was discovered, the simple main effects were analyzed. If no interaction was discovered, then the main effects were analyzed.

## 3 Results

### 3.1 Experimental design

We conducted a comparative study for the effects of two formulations of bupivacaine on peripheral nerve function in control and diabetic animals. A total of 7 animals were removed from analysis during the study period: 1 death, 3 did not meet the criteria for inclusion in the study (glucose < 200 mg/dl), 2 failed sciatic nerve block, and 1 sustained a foot injury from cage-mate. Analyses were performed on 33 control and 32 diabetic animals for all outcomes except locomotor behavior. Open-field analysis was completed for only one experimental cohort, including 12 control and 17 diabetic animals (Figure 1).

### 3.2 A high-fat diet and low-dose streptozotocin treatment successfully induce and maintain hyperglycemia and establish evoked responses consistent with peripheral neuropathy

#### 3.2.1 Body weight

**FIGURE 2:**
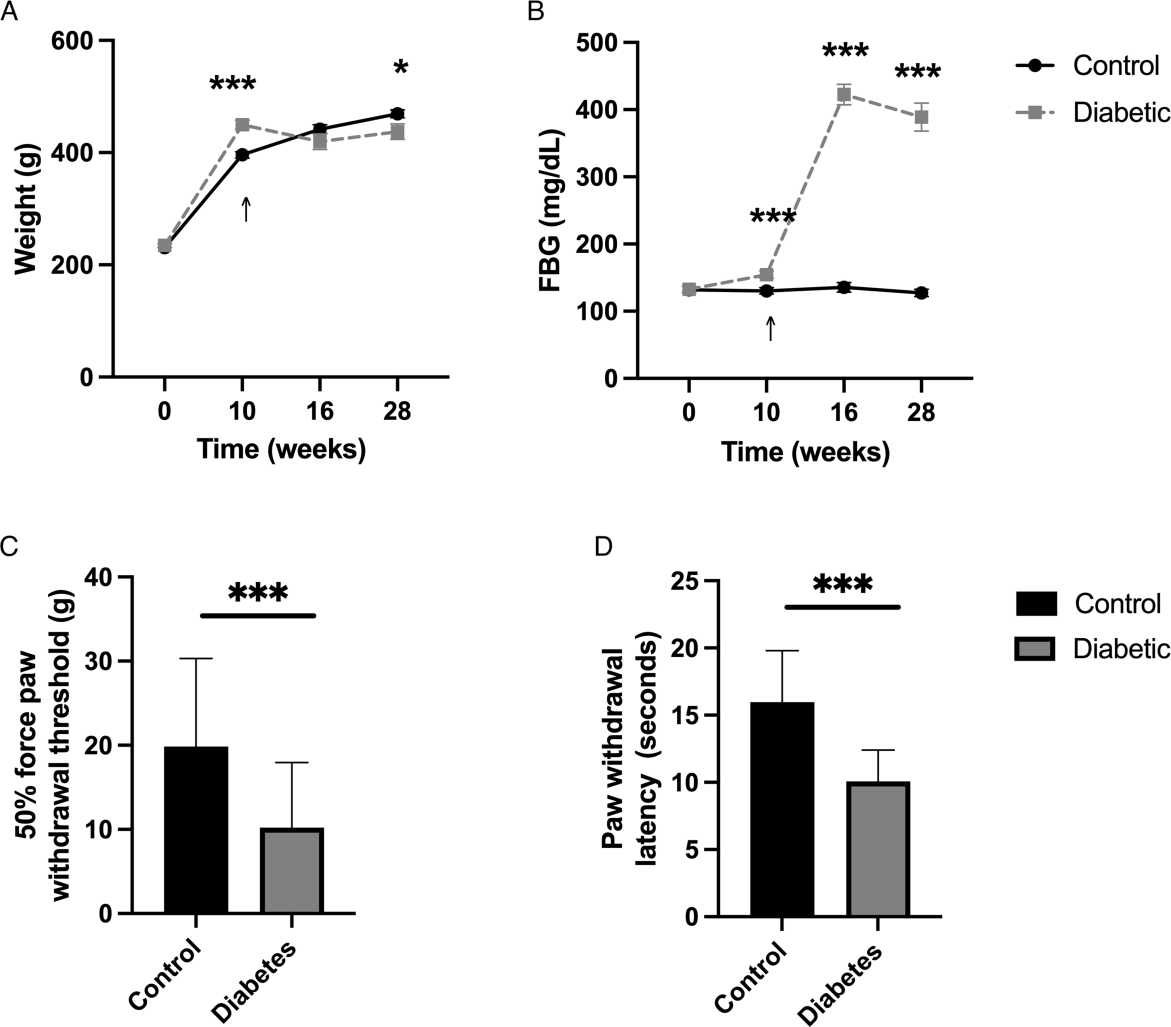
A high-fat diet and low-dose streptozotocin successfully induce and maintain hyperglycemia and establish evoked responses consistent with peripheral neuropathy. (**A**) Body weight and (**B**) fasting blood glucose (FBG) of control and diabetic animals at baseline (week 0), after 10 weeks of diet (week 10), six weeks after streptozotocin/vehicle injection (week 16), and 12 weeks after sciatic nerve blocks (week 28), show sustained differences consistent with diabetes. (**C**) Tactile and (**D**) thermal sensitivity increased, as demonstrated by reduced withdrawal threshold and latency in the diabetic animals compared to control animals six weeks after streptozotocin/vehicle injection (week 16). Tactile sensitivity was measured as a 50% force paw withdrawal threshold to von Frey filaments. Thermal sensitivity was measured as hind paw withdrawal latency to infrared heat stimulus. Arrows signify the time of streptozotocin/vehicle injection. Data expressed as mean ± SEM; *p < 0.05, **p < 0.01, *** *p* < 0.001.

Body weight was compared between control and diabetic groups throughout the study. At baseline (week 0), the mean body weight of the control and diabetic groups were similar (*p* = 0.330). After 10 weeks on assigned diets, the mean body weight of the diabetic group was statistically heavier compared to the control group (***p < 0.001). Six weeks after treatment with streptozotocin or vehicle (week 16), the mean body weight of the control and diabetic groups was again similar (*p* = 0.150). At the end of the experimental timeline (week 28), the mean body weight of the diabetic group was statistically lower compared to the control group (*p < 0.050) (Figure 2A; Supplementary Table 1A).

#### 3.2.2 Fasting blood glucose

Fasting blood glucose (FBG) was compared between control and diabetic groups throughout the study. At baseline (week 0), the mean FBG level of the control and diabetic groups were similar ( *p* = 0.910). After 10 weeks on assigned diets, the mean FBG level of the diabetic group was significantly greater than the control group (***p = 0.001). Six weeks after streptozotocin treatment (week 16), the mean FBG level was significantly higher in the diabetic group compared to the vehicle-treated control group (***p < 0.001). At the end of the experimental timeline (week 28), the mean FBG level of the diabetic group remained significantly higher compared to the control group (***p < 0.001) (Figure 2B; Supplementary Table 1B).

#### 3.2.3 Tactile allodynia and thermal hyperalgesia

Tactile allodynia and thermal hyperalgesia were compared between control and diabetic groups six weeks after hyperglycemia induction (week 16) using the von Frey and Hargreaves methods, respectively. The 50% force paw withdrawal threshold was significantly decreased in the diabetic group, suggesting the successful development of tactile allodynia [*t*(63) = 4.2, ***p < 0.001]. Similarly, the mean hind paw withdrawal latency after thermal stimulation via the Hargreaves test was significantly decreased in the diabetic group, suggesting the successful development of thermal hyperalgesia [*t*(63) = 7.45, ***p < 0.001] (Figures 2C,D; Supplementary Table 1C).

### 3.3 Twelve weeks after repeated sciatic nerve blocks, diabetic and bupivacaine-treated animals exhibited peripheral neuropathy based on evoked response measures

**FIGURE 3:**
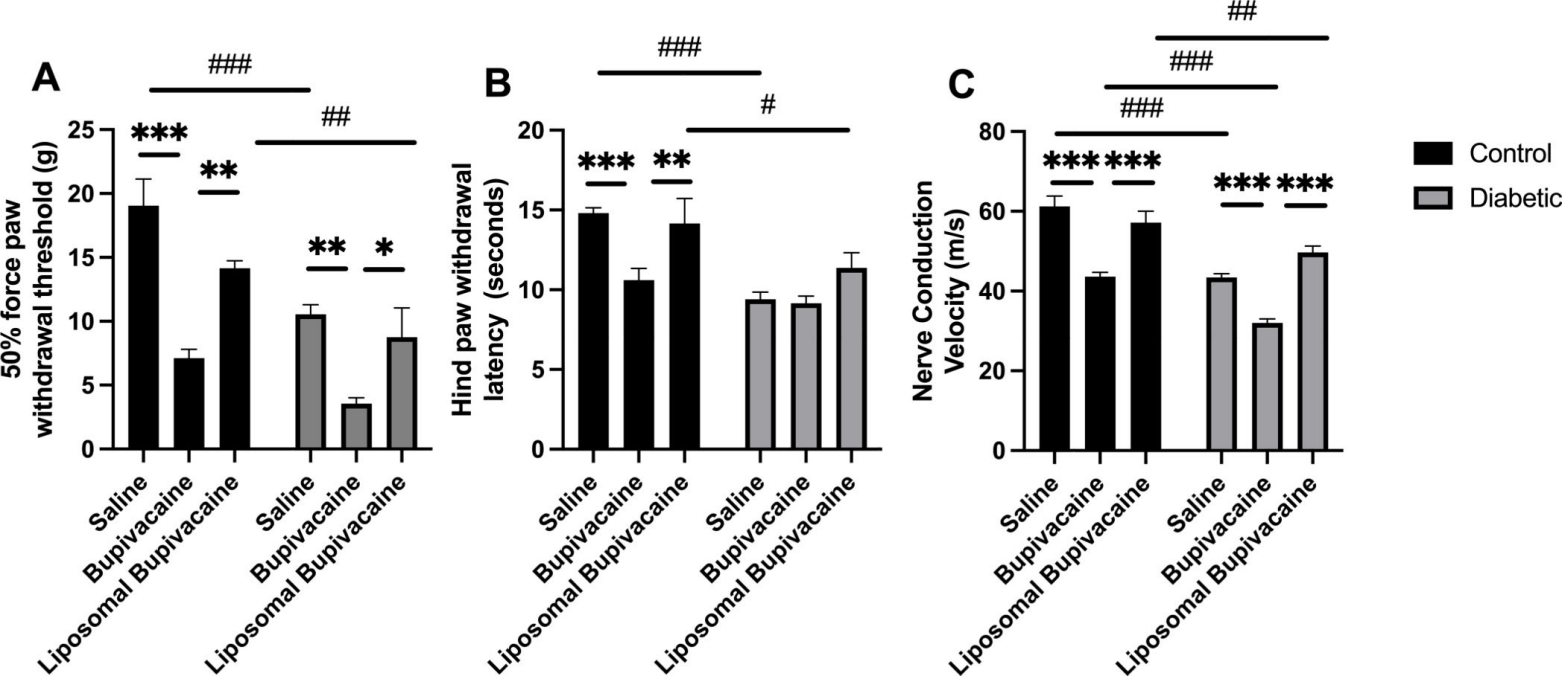
Twelve weeks after repeated sciatic nerve blocks, diabetic and bupivacaine-treated animals exhibited peripheral neuropathy based on evoked response measures. (**A**) Tactile sensitivity, (**B**) thermal sensitivity, and (**C**) nerve conduction velocity (NCV) were significantly worse in diabetic and/or bupivacaine-treated animals. Data expressed as mean ± SEM; *p < 0.05, **p < 0.01, ***p < 0.001 for within-group comparisons and ^#^*p* < 0.05, ^##^*p* < 0.01, ^###^*p* < 0.001 for between-group comparisons.

Twelve weeks after repeated sciatic nerve blocks, animals underwent a series of tests to evaluate evoked responses, including tactile allodynia, thermal hyperalgesia, and nerve conduction velocity (Figure 3). The means and standard error of the means for these responses are presented in Supplementary Table 2A, 2-way ANOVA results are presented in Supplementary Table 2B, and multiple comparisons results are presented in Supplementary Tables 2C–E.

#### 3.3.1 Tactile allodynia

There was no significant interaction between diabetic status and local anesthetic treatment, *F*(2,59) = 1.53, *p* = 0.226. However, there was a significant main effect for diabetic status *F*(2,59) = 24.3, ***p < 0.001, and local anesthetic treatment *F*(2,59) = 23.3, ***p = 0.001. Post hoc multiple comparisons indicated that bupivacaine treatment resulted in a significantly lower 50% force paw withdrawal threshold compared to saline in both control (***p < 0.001) and diabetic (**p = 0.004) animals (see Supplementary Table 2C for within and between group comparisons). Similarly, bupivacaine treatment resulted in a significantly lower 50% force paw withdrawal threshold compared to liposomal bupivacaine in both control (**p = 0.004) and diabetic (*p = 0.028) animals. There was no difference between saline and liposomal bupivacaine treatments for the control or diabetic groups. Between-group comparisons indicated that diabetic animals had a significantly lower 50% force paw withdrawal threshold compared to control animals for saline (^###^*p* < 0.001) and liposomal bupivacaine (^##^*p* = 0.014) treatments. There was no difference between control and diabetic animals treated with bupivacaine. Taken together, tactile allodynia was worse in diabetic animals overall, and bupivacaine treatment worsened tactile allodynia in both control and diabetic animals (Figure 3A; Supplementary Tables 2A–C).

#### 3.3.2 Thermal hyperalgesia

There was a significant interaction between diabetic status and local anesthetic treatment for hind paw withdrawal latency, *F*(2,59) = 3.2, *p = 0.048. A simple main effect analysis between local anesthetic treatments was significant for control animals but not diabetic animals. A *post hoc* multiple comparisons analysis demonstrated that bupivacaine-treated control animals had significantly faster mean hind paw withdrawal latency compared to saline-treated control animals (***p < 0.001), and liposomal bupivacaine-treated control animals (**p = 0.010). There was no significant difference between saline and liposomal treatments. There were no significant differences in mean hind paw withdrawal latency for diabetic animals treated with saline, bupivacaine, or liposomal bupivacaine. Simple main effects analysis between control and diabetic animals for each local anesthetic indicated a significant difference in the mean hind paw withdrawal latency for saline and liposomal bupivacaine treatment but not for treatment with bupivacaine. The *post hoc* multiple comparisons analysis demonstrated that the hind paw withdrawal latency was faster in diabetic animals treated with saline (^###^*p* < 0.001) and liposomal bupivacaine (^#^*p* = 0.022) compared to control animals. There was no difference between diabetic and control animals treated with bupivacaine. Taken together, thermal hyperalgesia was worse in the diabetic groups and bupivacaine-treated control animals (Figure 3B; Supplementary Tables 2A, 2B, 2D).

#### 3.3.3 Nerve conduction velocity

There was a significant interaction between diabetic status and local anesthetic treatment for the mean nerve conduction velocity, *F*(2,59) = 3.9, *p = 0.027. A simple main effect analysis between local anesthetic treatments for control and diabetic animals indicated a significant difference in the mean nerve conduction velocity for control and diabetic animals. A *post hoc* multiple comparisons analysis demonstrated that bupivacaine-treated control animals had significantly slower nerve conduction velocity compared to saline-treated control animals (***p < 0.001) and liposomal bupivacaine-treated control animals, (***p < 0.001). There was no significant difference between saline and liposomal treatments in control animals. Similarly, bupivacaine-treated diabetic animals had significantly slower nerve conduction velocity compared to saline-treated diabetic animals (***p < 0.001) and liposomal bupivacaine-treated diabetic animals (***p < 0.001). There was no significant difference between saline and liposomal treatments in diabetic animals. A simple main effect analysis between control and diabetic animals for each local anesthetic treatment indicated a significant difference in the mean nerve conduction velocity for saline, bupivacaine, and liposomal bupivacaine. The nerve conduction velocity was slower in diabetic animals treated with saline (^###^*p* < 0.001), bupivacaine (^###^*p* < 0.001), and liposomal bupivacaine (^##^*p* = 0.007), compared to control animals. Taken together, NCV was slower in diabetic and bupivacaine-treated animals. Notably, bupivacaine treatment in diabetic animals further exacerbated NCV (Figure 3C; Supplementary Tables 2A, B, E).

### 3.4 Twelve weeks after repeated sciatic nerve blocks, diabetic and bupivacaine-treated animals exhibited differences in exploratory and locomotor activities

**FIGURE 4:**
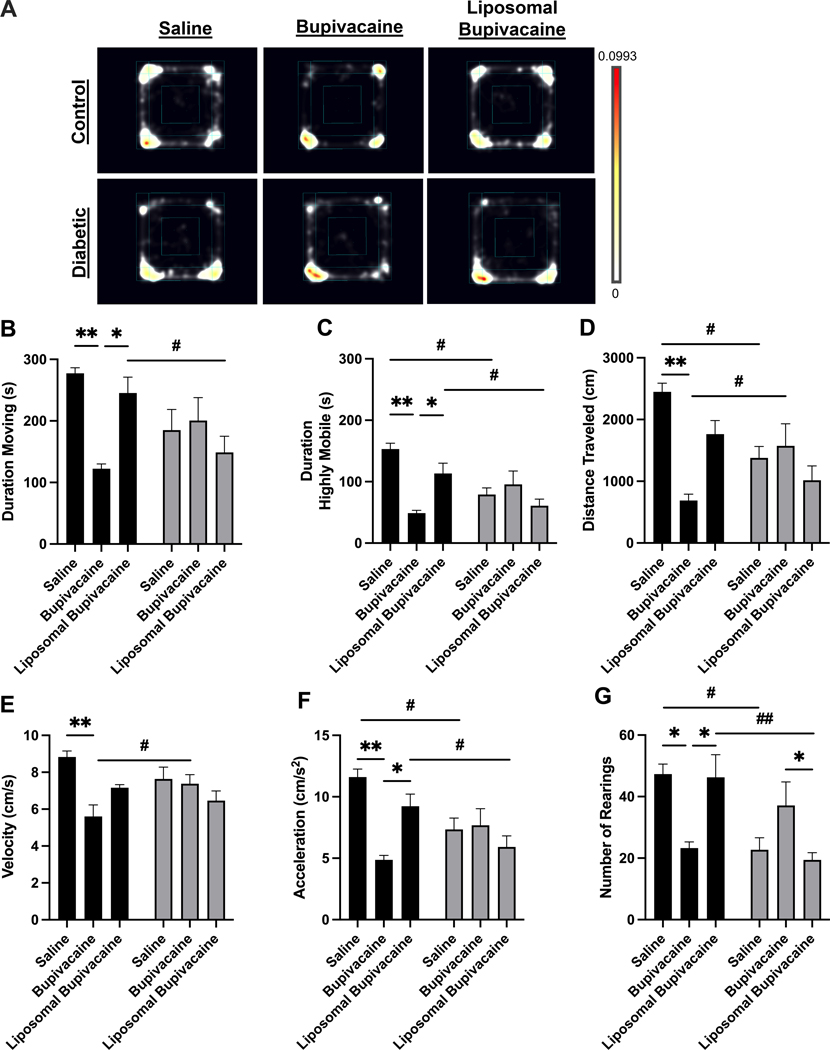
Twelve weeks after repeated sciatic nerve blocks, diabetic and bupivacaine-treated animals exhibited differences in exploratory and locomotor activities. General and kinematic activity was measured during 10 min of open field testing. (**A**) Heatmap representing relative dwell time in a given area for each treatment group. NodusEthoVision XT 15 software was used to analyze (**B**) duration moving, (**C**) duration spent highly mobile, (**D**) total distance traveled, (**E**) velocity, (**F**) acceleration, and (**G**) number of rears. Data expressed as mean ± SEM; *p < 0.05, **p < 0.01, ***p < 0.001 for within-group comparisons and ^#^*p* < 0.05, ^##^*p* < 0.01, ^###^*p* < 0.001 for between-group comparisons.

Twelve weeks after repeated sciatic nerve block, general and kinematic activity was measured during 10 min of open field testing (Figure 4; Supplementary Tables 3A–H). These measures included duration spent moving, duration spent highly mobile, distance traveled, mean velocity, mean acceleration, and number of rears. Descriptive statistics, ANOVA, and multiple comparison analyses for these measures are presented in Supplementary Tables 3A–E. A heatmap is presented in Figure 4A as a qualitative representation of location preferences for each treatment group. The colors show the proportion of time animals spent in a given area, averaged over all animals in a treatment group. Qualitatively, diabetic animals appear to spend less proportion of time exploring, as evidenced by color density being weighted on a particular side of the box or corner. Similarly, bupivacaine-treated control animals appear to spend less proportion of time exploring.

#### 3.4.1 Duration spent moving

There was a significant interaction between the diabetic status and local anesthetic treatment on time spent moving in the Open Field Arena, *F*(2, 23) = 5.3, *p = 0.013. A simple main effect analysis between local anesthetic treatments for control and diabetic animals indicated a significant difference in duration spent moving in control animals but not diabetic animals. A *post hoc* multiple comparisons analysis demonstrated that bupivacaine-treated control animals spent significantly less time moving compared to saline-treated (**p = 0.010) and liposomal bupivacaine-treated (*p = 0.049) control animals. There was no significant difference between saline and liposomal treatments in control animals. Furthermore, there were no significant differences in the duration spent moving between saline, bupivacaine, or liposomal bupivacaine treatments in diabetic animals. A simple main effect analysis between control and diabetic animals for each local anesthetic indicated a significant difference in duration spent moving for liposomal bupivacaine treatment only. A *post hoc* multiple comparisons analysis demonstrated that diabetic animals treated with liposomal bupivacaine spent less time moving compared to control animals treated with liposomal bupivacaine (^#^*p* = 0.031). There were no significant differences between control and diabetic animals treated with saline or bupivacaine. Taken together (Figure 4B; Supplementary Tables 3A–C).

#### 3.4.2 Duration spent highly mobile

There was a significant interaction between the diabetic status and local anesthetic treatment on time spent being highly mobile, *F*(2, 23) = 7.3, **p = 0.004. A simple main effect analysis between local anesthetic treatments for control and diabetic animals indicated a significant difference in duration spent highly mobile in control animals but not diabetic animals. A *post hoc* multiple comparisons analysis demonstrated that bupivacaine-treated control animals spent significantly less time being highly mobile compared to saline-treated (***p = 0.001) and liposomal bupivacaine-treated (*p = 0.049) control animals. There was no significant difference between saline and liposomal treatments in control animals. There were no significant differences in the duration spent highly mobile between saline, bupivacaine, or liposomal bupivacaine treatment in diabetic animals. A simple main effects analysis between control and diabetic animals for each local anesthetic indicated a significant difference in time spent highly mobile for saline, bupivacaine, and liposomal bupivacaine treatments. A *post hoc* multiple comparisons analysis demonstrated that control animals spent more time highly active compared to diabetic animals treated with saline (^#^*p* = 0.013) and liposomal bupivacaine (^#^*p* = 0.030). There was no significant difference between control and diabetic animals treated with bupivacaine (Figure 4C; Supplementary Tables 3A, B, D).

#### 3.4.3 Total distance traveled

There was a significant interaction between the diabetic status and local anesthetic treatment on total distance traveled, *F*(2, 23) = 6.6, **p = 0.005. A simple main effect analysis between local anesthetic treatments indicated a significant difference in the distance traveled for control animals but not diabetic animals. A *post hoc* multiple comparisons analysis demonstrated that bupivacaine-treated control animals traveled significantly less distance compared to saline-treated control animals (**p = 0.002). However, there were no differences between bupivacaine and liposomal bupivacaine or saline and liposomal bupivacaine treatments in control animals. There were no significant differences between local anesthetic treatments in diabetic animals. A simple main effect analysis between control and diabetic animals for each local anesthetic treatment indicated a significant difference in the distance traveled for saline and bupivacaine but not liposomal bupivacaine treatments. A *post hoc* multiple comparisons analysis demonstrated that diabetic animals traveled less distance if treated with saline (^#^*p* = 0.033). Interestingly, diabetic animals treated with bupivacaine traveled *more* distance (^##^*p* = 0.032) compared to control animals treated with bupivacaine. There was no significant difference in distance traveled between control and diabetic animals treated with liposomal bupivacaine (Figure 4D; Supplementary Tables 3A, B, E).

#### 3.4.4 Mean velocity

There was a significant interaction between the diabetic status and local anesthetic treatment on the mean velocity of animals moving in the open field arena, *F*(2,23) = 4.4, *p = 0.024. A simple main effect analysis between local anesthetic treatments for control animals indicated a significant difference in mean velocity. A *post hoc* multiple comparisons analysis demonstrated that bupivacaine-treated control animals had a slower mean velocity compared to saline-treated control animals (**p = 0.002). However, there were no significant differences between saline and liposomal treatments or bupivacaine and liposomal bupivacaine treatments in control animals. There were no significant differences in the mean velocity for diabetic animals treated with saline, bupivacaine, or liposomal bupivacaine. A simple main effect analysis between control and diabetic animals for each local anesthetic indicated a significant difference in mean velocity for bupivacaine but not saline or liposomal bupivacaine treatments. A *post hoc* multiple comparisons analysis demonstrated that control animals treated with bupivacaine traveled faster than diabetic animals treated with bupivacaine (^#^*p* = 0.021). There were no significant differences in mean velocity between control and diabetic animals treated with saline or liposomal bupivacaine (Figure 4E; Supplementary Tables 3A, B, F).

#### 3.4.5 Mean acceleration

There was a significant interaction between the diabetic status and local anesthetic treatment on the mean acceleration of animals moving in the open field arena, *F*(2,23) = 5.8, **p = 0.009. A simple main effect analysis between local anesthetic treatments for control animals indicated a significant difference in mean acceleration. A *post hoc* multiple comparisons analysis demonstrated that bupivacaine-treated control animals had a slower mean velocity compared to saline-treated control animals (**p = 0.002). However, there were no significant differences between saline and liposomal treatments or bupivacaine and liposomal bupivacaine treatments in control animals. There were no significant differences in the mean acceleration for diabetic animals treated with saline, bupivacaine, or liposomal bupivacaine. A simple main effect analysis between control and diabetic animals for each local anesthetic indicated a significant difference in mean acceleration for saline and liposomal bupivacaine but not bupivacaine treatments. A *post hoc* multiple comparisons analysis demonstrated that diabetic animals had lower acceleration compared to control animals when treated with saline (^#^*p* = 0.030), or liposomal bupivacaine (^#^*p* = 0.040). There was no significant difference in acceleration between control and diabetic animals treated with bupivacaine (Figure 4F; Supplementary Tables 3A, B, G).

#### 3.4.6 Rearing behavior

There was a significant interaction between the diabetic status and local anesthetic treatment on the average number of rears, *F*(2,23) = 8.1, **p = 0.002. A simple main effect analysis between local anesthetic treatments indicated a significant difference in the number of rears in both control and diabetic animals. A *post hoc* multiple comparisons analysis demonstrated that bupivacaine-treated control animals had fewer rears compared to saline-treated control animals (*p = 0.037), and liposomal bupivacaine-treated control animals (*p = 0.048). However, there was no significant difference between saline and liposomal treatments in control animals. In addition, bupivacaine-treated diabetic animals had fewer rears compared to liposomal bupivacaine-treated diabetic animals (*p = 0.043). There were no significant differences between saline and bupivacaine or between saline and liposomal bupivacaine-treated diabetic animals. A simple main effect analysis between control and diabetic animals for each local anesthetic indicated a significant difference in mean acceleration for saline and liposomal bupivacaine but not bupivacaine treatments. A *post hoc* multiple comparisons analysis demonstrated that diabetic animals had fewer rears compared to control animals when treated with saline (^#^*p* = 0.017), or liposomal bupivacaine (^##^*p* = 0.002). There was no significant difference in the number of rears between control and diabetic animals treated with bupivacaine (Figure 4G; Supplementary Tables 3A, B, H).

## 4 Discussion

Given the poor clinical sequelae related to progressive diabetic peripheral neuropathy, practitioners should consider the long-term consequences of peripheral nerve blocks using known toxic agents. Our goals are to identify neurotoxic risks associated with perineural local anesthetics and understand the mechanisms of local anesthetic toxicity to better inform the development of optimized dosing or agents that can reduce long-term dysfunction in this ever-increasing patient population with diabetes. Here, we tested the hypothesis that perineural bupivacaine would exacerbate the severity of sensorimotor dysfunction in high-fat diet/low-dose streptozotocin-treated animals. We first established that the high-fat diet/low-dose streptozotocin model of diabetes resulted in persistent hyperglycemia for the extended study period. Next, we showed peripheral neuropathy as evidenced by tactile allodynia and thermal hyperalgesia in diabetic animals. Finally, we performed unilateral sciatic nerve blocks using saline, bupivacaine, or liposomal bupivacaine on control and diabetic animals and evaluated evoked sensorimotor functions and general exploratory and locomotor behavior twelve weeks later. Our data demonstrate the negative effects of perineural bupivacaine on all sensorimotor functions tested in both control and diabetic animals. Furthermore, bupivacaine treatment in diabetic animals with pre-existing neuropathy caused continued worsening of sensorimotor function in some cases. These findings address concerns consistently raised in the literature regarding the safety of local anesthetic nerve blocks in patients with at-risk nerves and provide further evidence that bupivacaine has negative effects in both control and diabetic animals (5, 13–15, 22, 29, 30, 39–44). We describe long-term sensory and locomotor deficits in both control and diabetic animals after a sciatic nerve block using bupivacaine as compared to saline and liposomal bupivacaine.

The high-fat diet plus low-dose streptozotocin rodent model is a more recent model used to mimic the clinical condition of type 2 diabetes (45–47). Although no animal model is perfect, the high-fat diet/low-dose streptozotocin model of type 2 diabetes is now the most commonly used and best characterized, lending its value to testing functional outcomes of local anesthetic toxicity in regional anesthesia (39, 45–50). In line with others, our data show that animals fed a high-fat diet for 10 weeks initially increased their body weight compared to the control-fed animals. After treatment with low-dose streptozotocin, diabetic animals slowly decrease their body weight over time, presumably from the development of insulin resistance, as previously reported by others (51, 52). Few studies have evaluated this diabetic model at prolonged time points. While one study followed their model for 56 weeks, they repeated the streptozotocin injection every six weeks to maintain the hyperglycemic phenotype (53). To ensure the stability of this model for our experiments, we measured fasting blood glucose levels throughout the study period. Our data demonstrate that a single low-dose injection of streptozotocin is sufficient to induce sustained hyperglycemia at least up to 18 weeks after injection. Finally, our data confirmed the presence of both tactile and thermal allodynia in diabetic animals six and 18 weeks after streptozotocin treatment, supporting previously reported development of peripheral neuropathy in this rodent model (39, 47–49).

After confirming our experimental group had a phenotype consistent with diabetes, we tested the long-term effects of performing sciatic nerve blocks with bupivacaine and liposomal bupivacaine on tactile allodynia, thermal hyperalgesia, nerve conduction velocity, and spontaneous locomotor activity. Bupivacaine concentration (0.5%) was chosen based on common clinical practice and used an equal volume of liposomal bupivacaine. Importantly, our data reveal that even in control animals, treatment with bupivacaine resulted in long-term tactile allodynia, thermal hyperalgesia, and slowed nerve conduction velocity, while liposomal bupivacaine did not. Additionally, control animals treated with bupivacaine were less mobile, moved slower, and reared less when compared to saline and liposomal bupivacaine treatment. Our data also show that diabetes led to behavioral and neurophysiological changes consistent with worsening function for all local anesthetic treatment groups. Less clear are the exacerbating effects of bupivacaine on diabetic nerves. In diabetic animals, bupivacaine treatment led to worse tactile allodynia and slower NCV when compared to diabetic animals treated with saline or liposomal bupivacaine. However, no evidence of exacerbated nerve injury was found in other outcomes tested. Given the significant negative effect of diabetes alone, our study is likely underpowered to detect additional exacerbation by bupivacaine.

The safety of peripheral nerve blocks in patients with diabetic peripheral neuropathy has been discussed repeatedly in the clinical literature (5, 13, 14, 25, 32, 54–56). Studies have demonstrated increased sensitivity and prolonged block duration in animals and patients with diabetic peripheral neuropathy (28), and pre-clinical models have demonstrated increased local anesthetic toxicity in rodent models of diabetes (25, 57, 58). There is a growing number of reports demonstrating the neurotoxic effects of bupivacaine (22, 25, 39, 59). Specifically, bupivacaine has been identified as an independent risk factor for paresthesia/dysesthesia after use (22). While the overall reported incidence of acute nerve dysfunction after a peripheral nerve block is low, there is no long-term clinical data regarding the onset and progression of peripheral neuropathy in diabetic patients exposed to regional anesthesia. This is likely because following long-term outcomes is difficult in a clinical setting, given slow disease progression, lack of long-term follow-up, and evolving clinical practices. To our knowledge, this is the first study to describe the long-term effects of bupivacaine and liposomal bupivacaine in an experimentally induced model of diabetes. Given the increasing number of patients with diabetes who are presenting for surgical procedures, future research should continue to optimize our approach toward improved short- and long-term outcomes.

The mechanism of bupivacaine toxicity is not completely understood. It is known that the toxic effects appear to be time- and concentration-dependent. To perform a peripheral nerve block, relatively large amounts of local anesthetics are deposited perineurally to generate a concentration gradient of local anesthetic into the nerve. Furthermore, some patients undergo repeated nerve blocks, sustained nerve blocks with nerve catheters, or are exposed to adjuvants to increase the duration of analgesia. For these reasons, we performed repeated nerve blocks using both a short- and long-acting formulation of bupivacaine. Interestingly, strong clinical suspicion for increased nerve injury risk has led The American Society for Regional Anesthesia to suggest that clinicians use lower concentrations, avoid prolonged exposure, or use alternative methods in “at-risk” patients (56). Liposomal bupivacaine is a currently approved formulation marketed to provide ease of use in a single-shot nerve block with prolonged analgesic effectiveness (60). Interestingly, efficacy studies comparing bupivacaine and liposomal bupivacaine have revealed that liposomal bupivacaine may be less toxic than standard bupivacaine (30). Previously, we have demonstrated that liposomal bupivacaine did not exacerbate motoneuron death or delay functional recovery after a peripheral nerve injury (23). In the present study, we similarly found that liposomal bupivacaine caused less evidence of peripheral neuropathy in both control and diabetic animals. However, these findings only suggest that the liposomal formulation of bupivacaine should be further studied. Perhaps the slow release of bupivacaine from a liposomal formulation may expose nerves to lower concentrations over a given time or result in lower systemic exposure. Future studies should continue to focus on understanding the mechanism(s) of local anesthetic neurotoxicity and optimizing agents used for peripheral nerve blocks.

Nonetheless, the present results must be interpreted cautiously, and several limitations must be addressed. First, the differences between rodents and humans in terms of diabetes and neuropathy are unknown. Although the injection of streptozotocin is a common model of diabetes in rats, one should be cautious when extrapolating these results to humans. We used the von Frey and Hargreaves methods as surrogates for pain behavior since they are currently the most widely used and published methods for quantifying the severity of allodynia and hyperalgesia. However, we recognize that these are reflexive pain behaviors, and better assessments are needed that can measure in a more ethological manner. To overcome this limitation, we used the open-field test on a small cohort of animals in the present study to observe locomotor behavior. While others have also used this outcome measure for pain-like behavior, its validity is unclear (61). Cho et al. (62) reported reduced rears and distance traveled in a high-dose STZ model of diabetic neuropathic pain in mice. Future studies will utilize additional assessments that may reflect functional and affective pain-related features such as grimace scales and/or burrowing behaviors.

We believe that peripheral nerve blocks should continue to be a therapeutic option for patients, including those with diabetic peripheral neuropathy; however, a better understanding of the long-term risks of functional impairments is needed. Despite decades of clinical practice, research has not kept pace with the science needed to establish long-term safety. There is abundant space for further research to better understand the safety of peripheral nerve blocks in patients with diabetic peripheral neuropathy. Future studies can help elucidate the mechanism of neurotoxicity from local anesthetics, impact of tissue injury at the time of nerve block (surgical procedure for which acute pain is being treated), role of sex differences and age, and test alternative therapies.

## Conclusion

This study revealed that bupivacaine has long-term negative effects on the development of tactile allodynia and thermal hyperalgesia and results in the slowing of nerve conduction velocity in both control and diabetic animals. It is premature to extrapolate these findings to clinical guidance; however, given the escalating growth of the diabetic patient population who are already vulnerable to perioperative complications and progressive debilitation, research addressing long-term consequences, mechanisms of toxicity, and search for less toxic peripheral nerve block techniques should continue to be prioritized.

## Supplementary Material

Supplemental Table 1

Supplemental Table 2

Supplemental Table 3

## Data Availability

The original contributions presented in the study are included in the article/Supplementary Material, further inquiries can be directed to the corresponding author.
