# Supplementary material for: Long-term sensorimotor changes after a sciatic nerve block with bupivacaine and liposomal bupivacaine in a high-fat diet/low-dose streptozotocin rodent model of diabetes
Source: Front Anesthesiol. Author manuscript; Available in PMC 2025 Mar 19. (PMC11922546; doi:10.3389/fanes.2024.1422353)
Supplement: Supplemental Table 1 [file NIHMS2027933-supplement-Supplemental_Table_1.pdf]

Supplemental Tables 1 – Descriptive Statistics

A. Body Weight (g)

**Disease group**	N	Baseline		Week 10		Week 16		Week 28	
		Mean	SEM	Mean	SEM	Mean	SEM	Mean	SEM
**Control**	33	230.7	2.4	396.2	5.8	442.0	7.6	469.3	7.1
**Diabetic**	32	234.7	3.2	449.9	7.9	419.6	13.6	437.5	13.7

B. Fasting Blood Glucose (mg/dL)

**Disease group**	N	Baseline		Week 10		Week 16		Week 28	
		Mean	SEM	Mean	SEM	Mean	SEM	Mean	SEM
**Control**	33	131.8	5.0	130.4	4.7	135.5	7.0	127.1	5.4
**Diabetic**	32	132.6	4.8	154.5	5.5	422.5	15.4	388.8	20.7

C. Tactile and Thermal - Week 16

**Disease group**	N	Tactile (g)		Thermal (s)	
		Mean	SEM	Mean	SEM
**Control**	33	19.8	1.8	16.0	0.67
**Diabetic**	32	10.2	1.4	10.1	0.41

standard error of the mean (SEM)
