# Supplementary material for: Long-term sensorimotor changes after a sciatic nerve block with bupivacaine and liposomal bupivacaine in a high-fat diet/low-dose streptozotocin rodent model of diabetes
Source: Front Anesthesiol. Author manuscript; Available in PMC 2025 Mar 19. (PMC11922546; doi:10.3389/fanes.2024.1422353)
Supplement: Supplemental Table 2 [file NIHMS2027933-supplement-Supplemental_Table_2.pdf]

Supplemental Tables 2: Evoked Responses – Week 28

A. Descriptive Statistics

**Disease group**	Local anesthetic treatment		Tactile (g)		Thermal (s)		NCV (m/s)	
		N	Mean	SEM	Mean	SEM	Mean	SEM
**Control**	Saline	12	19.1	2.1	14.8	0.3	61.2	2.6
	Bupivacaine	12	7.1	0.7	10.6	0.7	43.6	1.1
	Liposomal Bupivacaine	9	14.1	0.6	14.2	1.6	57.2	2.9
**Diabetic**	Saline	9	10.6	0.8	9.4	0.4	43.4	0.9
	Bupivacaine	12	3.5	0.5	9.2	0.5	32.0	1.0
	Liposomal Bupivacaine	11	8.8	2.3	11.4	1.0	49.7	1.6

grams (g), seconds (s), meters/second (m/s), standard error of the mean (SEM)

B. 2-way ANOVA

		Tactile					Thermal					Nerve Conduction Velocity				
Source	*df*	SS	MS	*F*	*p*	η	SS	MS	*F*	*p*	η	SS	MS	*F*	*p*	η
Diabetic Status (A)	1	541	541.3	24.3	<.001***	0.29	164.7	164.7	23.9	<.001***	0.29	2407.3	2407.3	67.6	<.001***	0.53
Local Anesthetic (B)	2	1036	518.0	23.3	<.001***	0.44	102.6	51.3	7.4	.001***	0.20	3415.0	1707.5	47.9	<.001***	0.62
A×B	2	68	34.0	1.53	.226	0.05	44.0	22.0	3.2	.048*	0.10	274.9	137.4	3.9	.027*	0.12
Error	59	1313	22.3				406.6	6.9				21.1.2	35.6			
Univariate Tests																
Saline	1	NA					149.8	149.8	21.7	.001***	0.27	1627.3	1627.3	45.7	<.001***	0.44
Bupivacaine	1						12.6	12.6	1.8	.181	0.03	806.2	806.2	22.6	<.001***	0.28
Liposomal Bupivacaine	1						38.3	38.3	5.6	.022*	0.09	273.9	273.9	7.7	.007**	0.12
Error	59						406.6					2101.2	35.6			
Control	2	NA					120.1	60.0	8.7	<.001***	0.23	2003.0	1001.5	28.1	<.001***	0.49
Diabetic	2						32.5	16.3	2.4	.103	0.07	1848.9	924.4	26.0	<.001***	0.47
Error	59						406.6	6.9				2101.2	35.6			

sum of squares (SS), mean of squares, (MS), F-test (*F*), probability value (*p*), effect size (η)

C. Multiple Comparisons – Tactile

**Disease group**	**Local anesthetic comparison**	Tactile (g)			
		Mean Difference	SE	*p*	95% CI Lower to Upper
**Control**	Saline-Bupivacaine	11.94	1.93	<.001***	7.31 to 16.57
	Saline-Liposomal Bupivacaine	4.90	2.08	.056	-0.98 to 9.91
	Bupivacaine-Liposomal Bupivacaine	-7.03	2.08	.004**	12.04 to -2.03
**Diabetic**	Saline-Bupivacaine	7.01	2.08	.004**	2.01 to 12.01
	Saline-Liposomal Bupivacaine	1.80	2.12	.675	-2.30 to 6.90
	Bupivacaine-Liposomal Bupivacaine	-5.21	1.97	.028*	-9.94 to -0.47
**Local Anesthetic**	**Disease group comparison**	Mean Difference	SE	*p*	95% CI Lower to Upper
**Saline**	Control-Diabetic	8.50	2.08	<.001###	4.34 to 12.66
**Bupivacaine**	Control-Diabetic	3.57	1.93	.069	-0.29 to 7.42
**Liposomal Bupivacaine**	Control-Diabetic	5.39	2.12	.014#	1.15 to 9.64

standard error (SE), *p*-value (*p*), confidence interval (CI)

D. Multiple Comparisons – Thermal

Disease group	Local anesthetic comparison	Thermal (s)			
		Mean Difference	SE	*p*	95% CI Lower to Upper
**Control**	Saline-Bupivacaine	4.21	1.07	<.001***	1.57 to 6.85
	Saline-Liposomal Bupivacaine	0.65	1.16	1.0	-2.2 to 3.51
	Bupivacaine-Liposomal Bupivacaine	-3.56	1.16	.01**	-6.41 to -0.77
**Diabetic**	Saline-Bupivacaine	0.26	1.16	1.0	-2.59 to 3.11
	Saline-Liposomal Bupivacaine	-1.96	1.18	.31	-4.87 to 0.95
	Bupivacaine-Liposomal Bupivacaine	-2.22	1.10	.14	-4.92 to 0.48

Local Anesthetic	Disease group comparison	Mean Difference	SE	*p*	95% CI Lower to Upper
**Saline**	Control-Diabetic	5.40	1.16	<.001###	3.08 to 7.71
**Bupivacaine**	Control-Diabetic	1.45	1.07	.181	-0.69 to 3.60
**Liposomal Bupivacaine**	Control-Diabetic	2.78	1.18	.022#	0.42 to 5.14

E. Multiple Comparisons – Nerve Conduction Velocity

Disease group	Local anesthetic comparison	Nerve Conduction Velocity (m/s)			
		Mean Difference	SE	*p*	95% CI Lower to Upper
**Control**	Saline-Bupivacaine	17.59	2.43	<.001***	11.59 to 23.60
	Saline-Liposomal Bupivacaine	4.07	2.63	.382	-2.42 to 10.55
	Bupivacaine-Liposomal Bupivacaine	-13.52	2.63	<.001***	-20.01 to -7.04
**Diabetic**	Saline-Bupivacaine	11.40	2.63	<.001***	4.91 to 17.88
	Saline-Liposomal Bupivacaine	-6.28	2.68	.068	-12.89 to 0.33
	Bupivacaine-Liposomal Bupivacaine	-17.68	2.49	<.001***	-23.81 to -11.54
**Local Anesthetic**	**Disease group comparison**	Mean Difference	SE	*p*	95% CI Lower to Upper
**Saline**	Control-Diabetic	17.89	2.63	<.001###	12.52 to 23.05
**Bupivacaine**	Control-Diabetic	11.59	2.44	<.001###	6.72 to 16.47
**Liposomal Bupivacaine**	Control-Diabetic	7.44	2.68	.007##	2.07 to 12.81
