# Supplementary material for: Long-term sensorimotor changes after a sciatic nerve block with bupivacaine and liposomal bupivacaine in a high-fat diet/low-dose streptozotocin rodent model of diabetes
Source: Front Anesthesiol. Author manuscript; Available in PMC 2025 Mar 19. (PMC11922546; doi:10.3389/fanes.2024.1422353)
Supplement: Supplemental Table 3 [file NIHMS2027933-supplement-Supplemental_Table_3.pdf]

Supplemental Tables 3 – Open Field Tests
A. Descriptive Statistics

**Disease group**	**Local anesthetic treatment**		Duration moving (s)		Duration highly mobile (s)		Distance traveled (cm)		Velocity (cm/s)		Acceleration (cm/s^2)		Number of rears	
		N	Mean	SEM	Mean	SEM	Mean	SEM	Mean	SEM	Mean	SEM	Mean	SEM
**Control**	Saline	4	277.4	8.9	153.1	9.6	2448	140	8.8	0.32	11.61	0.64	47.3	3.3
	Bupivacaine	4	122.5	7.8	48.8	4.5	689	103	5.6	0.63	4.87	0.37	23.3	2.0
	Liposomal Bupivacaine	4	245.4	25.6	113.3	16.9	1763	221	7.2	0.17	9.23	1.0	46.3	7.4
**Diabetic**	Saline	3	185.2	33.5	79.0	10.9	1379	184	7.6	0.64	7.34	0.92	22.7	3.9
	Bupivacaine	7	200.6	37.2	95.7	21.7	1574	357	7.4	0.49	7.68	1.4	37.1	7.6
	Liposomal Bupivacaine	7	148.9	26.3	60.8	10.8	1015	234	6.5	0.53	5.91	0.91	19.4	2.3

B. 2-way ANOVA

Source	*df*	Duration moving					Duration highly mobile					Distance traveled				
Source	*df*	SS	MS	*F*	*p*	η	SS	MS	*F*	*p*	η	SS	MS	*F*	*p*	η
Diabetic Status (A)	1	8937	8937	2.0	.172	0.08	4631	4631	3.5	.073	0.13	634000	634000	1.7	.211	0.07
Local Anesthetic (B)	2	20316	10158	2.3	.127	0.16	7915	3958	3.0	.069	0.21	2529092	126456	3.3	.055	0.22
A×B	2	47675	23837	5.3	$.013^{*}$	0.32	19083	9542	7.3	$.004^{**}$	0.39	5053477	2526739	6.6	$.005^{**}$	0.37
Error	23	103491	4500				30165	1312				8810274	383055			
Univariate Tests																
Saline	1	14567	14567	3.2	.085	0.12	9406	9406	7.2	$.013^{*}$	0.24	1960540	1960540	5.1	$.033^{*}$	0.18
Bupivacaine	1	15542	15542	3.5	.076	0.13	5599	5599	4.3	$.050^{*}$	0.16	1994202	1994202	5.2	$.032^{*}$	0.19
Liposomal Bupivacaine	1	23739	23739	5.3	$.031^{*}$	0.19	7004	7004	5.3	$.030^{*}$	0.19	1421744	1421744	3.7	.066	0.14
Control	2	53538	26769	5.9	$.008^{\#\#}$	0.34	22160	11080	8.4	$.002^{\#\#}$	0.42	6293141	3146571	8.2	$.002^{\#\#}$	0.42
Diabetic	2	9636	4818	1.1	.359	0.09	4261	2131	1.6	.219	0.12	1109790	554890	1.4	.256	0.11
Error	23	103491	4500				30165	1312				8810274	383055			
		Velocity					Acceleration					Number of rears				
Diabetic Status (A)	1	0.009	0.009	0.007	.934	0	16.6	16.6	2.8	.106	0.11	1028	1028	6.6	$.017^{*}$	0.22
Local Anesthetic (B)	2	13.4	6.7	5.1	$.015^{*}$	0.31	41.9	21.0	3.6	$.044^{*}$	0.24	96	48	0.3	.738	0.03
A×B	2	11.7	5.8	4.4	$.024^{*}$	0.28	68.5	34.2	5.8	$.009^{**}$	0.34	2531	1265	8.1	$.002^{**}$	0.41
Error	23	30.4	1.3				134.8	5.9				3589	156			
Univariate Tests																
Saline	1	2.4	2.4	1.8	.187	0.07	31.3	31.3	5.3	$.030^{*}$	0.19	1036	1036	6.6	.017*	0.22
Bupivacaine	1	8.1	8.1	6.1	$.021^{*}$	0.21	20.1	20.1	3.4	.077	0.13	491	491	3.1	.089	0.12
Liposomal Bupivacaine	1	1.3	1.3	0.9	.341	0.04	27.9	27.9	4.8	$.040^{*}$	0.17	1831	1831	11.7	.338	0.34
Control	2	20.9	10.4	7.9	$.002^{\#\#}$	0.41	93.5	46.7	8.0	$.002^{\#\#}$	0.41	1474	737	4.7	$.019^{\#}$	0.29
Diabetic	2	4.3	2.1	1.6	.221	0.12	11.7	5.8	1.0	.384	0.08	1176	588	3.8	$.038^{\#}$	0.25
Error	23	30.4	1.3				134.8	5.9				3589	156			

C. Multiple Comparisons – Duration Moving

Disease group	Local anesthetic comparison	Duration moving (s)			
		Mean Difference	SE	*p*	95% CI Lower to Upper
**Control**	Saline-Bupivacaine	154.9	47.4	.010**	32.5 to 277.4
	Saline-Liposomal Bupivacaine	32.0	47.4	1.0	-90.5 to 154.4
	Bupivacaine-Liposomal Bupivacaine	-123.0	47.4	.049*	-245.5 to -0.51
**Diabetic**	Saline-Bupivacaine	-15.4	46.3	1.0	-134.9 to 104.1
	Saline-Liposomal Bupivacaine	36.4	46.3	1.0	-83.2 to 155.9
	Bupivacaine-Liposomal Bupivacaine	51.7	35.9	.488	-40.9 to 144.3
**Local Anesthetic**	**Disease group comparison**	Mean Difference	SE	*p*	95% CI Lower to Upper
**Saline**	Control-Diabetic	92.2	51.2	.085	-13.8 to 198.1
**Bupivacaine**	Control-Diabetic	-78.1	42.0	.076	-165.1 to 8.8
**Liposomal Bupivacaine**	Control-Diabetic	96.6	42.0	.031#	9.6 to 183.5

standard error (SE), probability value (*p*), confidence interval (CI)

D. Multiple Comparisons – Duration highly mobile

Disease group	Local anesthetic comparison	Duration highly mobile (s)			
		Mean Difference	SE	*p*	95% CI Lower to Upper
**Control**	Saline-Bupivacaine	104.2	25.6	.001***	38.2 to 170.4
	Saline-Liposomal Bupivacaine	39.8	25.6	.401	-26.3 to 105.9
	Bupivacaine-Liposomal Bupivacaine	-64.5	25.6	.058	-130.9 to 1.6
**Diabetic**	Saline-Bupivacaine	-16.7	25.0	1.0	-81.2 to 47.8
	Saline-Liposomal Bupivacaine	18.2	25.0	1.0	-46.3 to 82.7
	Bupivacaine-Liposomal Bupivacaine	34.9	19.4	.254	-15.1 to 84.9
**Local Anesthetic**	**Disease group comparison**	Mean Difference	SE	*p*	95% CI Lower to Upper
**Saline**	Control-Diabetic	74.1	27.7	.013#	16.9 to 131.3
**Bupivacaine**	Control-Diabetic	-46.9	22.7	.050	-93.9 to 0.05
**Liposomal Bupivacaine**	Control-Diabetic	52.5	22.7	.030#	-99.4 to -5.5

standard error (SE), probability value (*p*), confidence interval (CI)

E. Multiple Comparisons – Distance traveled

Disease group	Local anesthetic comparison	Distance traveled (cm)			
		Mean Difference	SE	p	95% CI Lower to Upper
**Control**	Saline-Bupivacaine	1759.7	437.6	.002**	629.7 to 2889.6
	Saline-Liposomal Bupivacaine	685.8	437.6	.392	-444.2 to 1815.8
	Bupivacaine-Liposomal Bupivacaine	-1073.9	437.6	.066	-2203.9 to 56.1
**Diabetic**	Saline-Bupivacaine	-194.9	427.1	1.0	-1297.6 to 907.9
	Saline-Liposomal Bupivacaine	363.7	427.1	1.0	-739.0 to 1466.4
	Bupivacaine-Liposomal Bupivacaine	558.6	330.8	.314	-295.6 to 1412.8
**Local Anesthetic**	**Disease group comparison**	Mean Difference	SE	p	95% CI Lower to Upper
**Saline**	Control-Diabetic	1069.4	472.7	.033#	91.6 to 2047.3
**Bupivacaine**	Control-Diabetic	-885.1	387.9	.032#	-1687.6 to -82.6
**Liposomal Bupivacaine**	Control-Diabetic	747.4	387.9	.066	-55.1 to 1549.8

standard error (SE), probability value (*p*), confidence interval (CI)

F. Multiple Comparisons - Velocity

**Disease group**	**Local anesthetic comparison**	Velocity (cm/s)			
		Mean Difference	SE	*p*	95% CI Lower to Upper
**Control**	Saline-Bupivacaine	3.2	0.8	.002**	1.1 to 5.3
	Saline-Liposomal Bupivacaine	1.7	0.8	.153	-0.4 to 3.8
	Bupivacaine-Liposomal Bupivacaine	-1.6	0.8	.204	-3.7 to 0.4
**Diabetic**	Saline-Bupivacaine	0.3	0.8	1.0	-1.8 to 2.3
	Saline-Liposomal Bupivacaine	1.2	0.8	.451	-0.9 to 3.2
	Bupivacaine-Liposomal Bupivacaine	0.9	0.6	.438	-0.7 to 2.5
**Local Anesthetic**	**Disease group comparison**	Mean Difference	SE	*p*	95% CI Lower to Upper
**Saline**	Control-Diabetic	1.2	0.9	.187	-0.6 to 3.0
**Bupivacaine**	Control-Diabetic	-1.8	0.7	.021#	-3.3 to -0.3
**Liposomal Bupivacaine**	Control-Diabetic	0.7	0.7	.341	-0.8 to 2.2

standard error (SE), probability value (*p*), confidence interval (CI)

G. Multiple Comparisons - Acceleration

Disease group	Local anesthetic comparison	Acceleration (cm/s^2)			
		Mean Difference	SE	*p*	95% CI Lower to Upper
**Control**	Saline-Bupivacaine	6.7	1.7	.002**	2.3 to 11.2
	Saline-Liposomal Bupivacaine	2.4	1.7	.530	-2.0 to 6.8
	Bupivacaine-Liposomal Bupivacaine	-4.4	1.7	.054	-8.8 to 0.07
**Diabetic**	Saline-Bupivacaine	-0.3	1.7	1.0	-4.7 to 4.0
	Saline-Liposomal Bupivacaine	1.4	1.7	1.0	-2.9 to 5.7
	Bupivacaine-Liposomal Bupivacaine	1.8	1.3	.554	-1.6 to 5.1
**Local Anesthetic**	**Disease group comparison**	Mean Difference	SE	*p*	95% CI Lower to Upper
**Saline**	Control-Diabetic	4.3	1.8	.030#	0.4 to 8.1
**Bupivacaine**	Control-Diabetic	-2.8	1.5	.077	-6.0 to 0.33
**Liposomal Bupivacaine**	Control-Diabetic	3.3	1.5	.040#	0.2 to 6.5

standard error (SE), probability value (*p*), confidence interval (CI)

H. Multiple Comparisons – Number of rears

Disease group	Local anesthetic comparison	Number of rears			
		MD	SE	*p*	95% CI Lower to Upper
**Control**	Saline-Bupivacaine	24.0	8.8	.037*	1.2 to 46.8
	Saline-Liposomal Bupivacaine	1.0	8.8	1.0	-21.8 to 23.8
	Bupivacaine-Liposomal Bupivacaine	-23.0	8.8	.048*	-45.8 to -0.2
**Diabetic**	Saline-Bupivacaine	-14.5	8.6	.320	-36.7 to 7.8
	Saline-Liposomal Bupivacaine	3.2	8.6	1.0	-19.0 to 25.5
	Bupivacaine-Liposomal Bupivacaine	17.7	6.7	.043*	0.5 to 35.0
**Local Anesthetic**	**Disease group comparison**	Mean Difference	SE	*p*	95% CI Lower to Upper
**Saline**	Control-Diabetic	24.6	9.5	.017#	4.8 to 44.3
**Bupivacaine**	Control-Diabetic	-13.9	7.8	.089	-30.1 to 2.3
**Liposomal Bupivacaine**	Control-Diabetic	26.8	7.8	.002##	10.6 to 43.0

standard error (SE), probability value (*p*), confidence interval (CI)
